# Feasibility and acceptability of ACT for the community case management of malaria in urban settings in five African sites

**DOI:** 10.1186/1475-2875-10-240

**Published:** 2011-08-16

**Authors:** Patricia Akweongo, Peter Agyei-Baffour, Morankar Sudhakar, Bertha N Simwaka, Amadou T Konaté, Philip B Adongo, Edmund NL Browne, Ayalew Tegegn, Doreen Ali, Abdoulaye Traoré, Mary Amuyunzu-Nyamongo, Franco Pagnoni, Guy Barnish

**Affiliations:** 1 Department of Policy, Planning and Management, School of Public Health, University of Ghana, P.O. Box LG13, Legon, Ghana; 2 Department of Community Health, School of Medical Sciences, KNUST, Kumasi, Ghana; 3 Health Education and Behavioural Sciences Department, Jimma University, P. O. Box - 378 Jimma, Ethiopia; 4 Improving Malaria Diagnostics Project, Research for Equity and Community Health Trust (REACH), P:O: Box 1597, Lilongwe, Malawi; 5 Centre National de Recherche et de Formation sur le Paludisme, 01 BP 2208, Ouagadougou 01, Burkina Faso; 6 Department of Epidemiology and Biostatistics, Jimma University, Jimma, Ethiopia; 7 Malawi National Malaria Control Program, Private Bag 65, Lilongwe, Malawi; 8 African Institute for Health and Development, P:O: Box 45259-00100, Nairobi, Kenya; 9 Evidence for Antimalarial Policy and Access Unit, UNICEF/UNDP/World Bank/WHO Special Programme for Research and Training in Tropical Diseases (TDR), Geneva, Switzerland; 10 le Moncheny, 23400 St. Moreil, Limousin, France

## Abstract

**Background:**

The community case management of malaria (CCMm) is now an established route for distribution of artemisinin-based combination therapy (ACT) in rural areas, but the feasibility and acceptability of the approach through community medicine distributors (CMD) in urban areas has not been explored. It is estimated that in 15 years time 50% of the African population will live in urban areas and transmission of the malaria parasite occurs in these densely populated areas.

**Methods:**

Pre- and post-implementation studies were conducted in five African cities: Ghana, Burkina Faso, Ethiopia and Malawi. CMDs were trained to educate caregivers, diagnose and treat malaria cases in < 5-year olds with ACT. Household surveys, focus group discussions and in-depth interviews were used to evaluate impact.

**Results:**

Qualitative findings: In all sites, interviews revealed that caregivers' knowledge of malaria signs and symptoms improved after the intervention. Preference for CMDs as preferred providers for malaria increased in all sites.

Quantitative findings: 9001 children with an episode of fever were treated by 199 CMDs in the five study sites. Results from the CHWs registers show that of these, 6974 were treated with an ACT and 6933 (99%) were prescribed the correct dose for their age. Fifty-four percent of the 3,025 children for which information about the promptness of treatment was available were treated within 24 hours from the onset of symptoms.

From the household survey 3700 children were identified who had an episode of fever during the preceding two weeks. 1480 (40%) of them sought treatment from a CMD and 1213 of them (82%) had received an ACT. Of these, 1123 (92.6%) were administered the ACT for the correct number of doses and days; 773 of the 1118 (69.1%) children for which information about the promptness of treatment was available were treated within 24 hours from onset of symptoms, and 768 (68.7%) were treated promptly and correctly.

**Conclusions:**

The concept of CCMm in an urban environment was positive, and caregivers were generally satisfied with the services. Quality of services delivered by CMDs and adherence by caregivers are similar to those seen in rural CCMm settings. The proportion of cases seen by CMDs, however, tended to be lower than was generally seen in rural CCMm. Urban CCMm is feasible, but it struggles against other sources of established healthcare providers. Innovation is required by everyone to make it viable.

## Background

Urbanization in Africa is increasing at such a rate that the United Nations estimates that 50% of the African population will live in urban areas by 2025 [1].

Since the launch of Primary Health Care (PHC) [2], the value of rural community volunteers providing early diagnosis and treatment of malaria has been recognized and documented. In the last 15 years, a number of projects have demonstrated that community-based interventions based on training caregivers (e.g. mothers) or community members to provide early diagnosis and treatment have great potential [3-7]. This led to the development of the WHO Home Management of Malaria (HMM) strategy in 2005 [8], which aims to improve access to effective treatment for uncomplicated malaria in children. HMM is currently adopted in 18 countries (Pagnoni, pers. comm.).

In general, the community providers are normally community volunteers, although shopkeepers have been used in Kenya [7,9]. Most recently, a change of name from HMM to community case-management of malaria (CCMm) has been proposed:

"Community Case Management of Malaria (CCMm) is the strategy recommended by WHO to improve access to prompt and effective treatment of malaria episodes through trained community members living as close as possible to where the patients live. This strategy was previously known as the HMM (which is now considered to be an inaccurate description of the implementation) due to the misinterpretation of the concept to imply availability of drugs freely in the home without a system for regulating use."

As originally developed, CCMm focused on rural, underserved areas with high malaria transmission in Africa. However, the on-going urbanization process and consequent epidemiological changes demand for an improvement of access to effective malaria treatment in urban settings [10], therefore, the possibility of implementing CCMm in urban settings needs to be explored. However, there are several factors that make urban contexts different from rural areas with regard to the community-case management of malaria. Urban areas have, in general, greater health provider diversity, are socio-economically and culturally more diverse, and operate under different governmental structures. Furthermore, the transmission pattern and intensity are often different from rural areas. Many of these factors influence health-seeking behaviour and perceptions about the disease, as well as case-definition and the epidemiology of malaria, and, in general, methods, points and approaches for potential intervention.

Health interventions delivered by community health workers are recognized as having the potential to accelerate progress in reaching the MDG in high mortality settings [11,12]. Concerns, however, have been expressed about the sustainability of their action [13] and about the appropriateness of employing CMDs to distribute ACT. Concerns are mainly related to the potential for poor adherence of treatment regimes by both the caregivers and the CMDs, which could lead to the development of drug resistance by the malaria parasites [14,15]. These fears, however, have now been partially overcome as CCMm has been shown to be a feasible and effective treatment option in rural settings [16,17], and is adopted as a malaria control strategy in several endemic countries.

To evaluate the feasibility of CCMm in urban settings in sub-Saharan Africa, the UNICEF-UNDP-WORLD BANK-WHO Special Programme for Research and Training in Tropical Diseases (TDR) funded studies in urban areas in Ghana, Ethiopia, Burkina Faso and Malawi. This paper reports on the effectiveness of conducting CCMm (directed specifically to under-5 year olds) in a variety of urban settings, representing different health systems and socio-cultural environments.

## Methods

### Study areas and populations

The studies were carried out in the African urban areas of Kumasi and Bolgatanga, Ghana; Jimma, Ethiopia; Lilongwe, Malawi, and Ouagadougou, Burkina Faso, selected by an advisory committee from those who responded to a call published by TDR in 2005.

Variations in the methodologies were unavoidable, since in each case the specific national health framework was followed. The characteristics of each study site are summarized in Table 1. In Kumasi, Ghana, ten urban and ten peri-urban communities within the Asokwa sub-metro (ward) were selected. There were one University Hospital, a Government Hospital, chemists, and 14 private hospitals, 22 private clinics, three Homeopathic clinics, one private laboratory and two mission clinics. All children aged 6-59 months and their caregivers and/or persons in the home who influence treatment-seeking behaviour were selected for the study. This high malaria prevalence area (37%) exhibits poor socio-economic settings with clearly identifiable cultural enclaves and poor access to health care. The study population of Bolgatanga (Ghana) was selected from the Central, Intermediate and Peripheral electoral groups of the township. Within this area there is a wide variety of health providers, including the Regional Hospital, a health centre, a poly-clinic, several clinics and a variety of private providers.

**Table 1:** Characteristics of the study sites

Kumasi	Bolgatanga	Jimma	Lilongwe	Ouagadougou
Population estimate: 1,529,151	Total population of metropolitan Bolgatanga estimated at 147,729	Population: 159,000	Population: 24,000	Population: 1.2 million (2006)

**Study conducted **in Asokwa sub-metro with a population of 566,980 (2005), which is 30.3% of the Kumasi population	**Study conducted **in the urban town (population 72,768)	**Study conducted **in whole town	**Study conducted **in Kauma, a squatter settlement within urban Lilongwe	**Study conducted **in adjacent districts of Wemtenga and Taabtenga: two adjoining and contrasting neighbourhood. Wemtenga was subdivided, built up and serviced with all the urban amenities, while peripheral Taabtenga was not subdivided and had a rural appearance

Malaria prevalence of 37% and accounts for over 45% out-patient cases	Malaria prevalence of 22%. Children < 5 years with uncomplicated malaria represented 29.5% of all reported cases in 2007	The peak of malaria incidence follows the main rainfall season in July, August and September. Prevalence < = 4.9% in the population, but was 20.43% in < 5 year-olds (2006)	Malaria prevalence in < 5 s was 14% (2010 Lilongwe malaria survey)	Case morbidity in under 5 years in high transmission season is approximately 50%
Children < 5 years and infants form 20% and 4%, respectively	Children < 5 years: 13% of the population	Children < 5 years: 9.2% of total population	Children < 5 years: 19% of the population	Children < 5 years: 18.8% of study population (1,970/10,500)

Main occupations are trading and farming Cultivation of vegetables and other crops is common	Presence of urban farming in immediate peri-urban areas	Many houses have gardens (bananas, coffee, kitchen gardens, etc)	Most people earn a living through petty vending or working as unskilled labourers while others (30%) are unemployed	Main occupations are: In Wemtenga: Salaried and trading In Taabtenga: farming, pretty vending and working as unskilled labourers.

Jimma has two public health centres and 13 private clinics, one Jimma University Specialized Hospital and 15 pharmacies. The peripheral area of the town is surrounded by swamps; lack of a proper drainage system, and two big streams containing sewerage water. The study participants were residents of 10 *kebeles *(the lowest administrative unit in Ethiopia).

Kauma, a squatter settlement within Lilongwe, Malawi has one of the highest poverty levels in an urban setting; no health facilities, and transport costs to the nearest health facility posed serious barriers. The caregivers and children were residents of the 24,000 population of this settlement.

In Ouagadougou, two adjacent districts were selected for the study: Wemtenga (served by modern health facilities) is a built up area, which is serviced with all the urban amenities, and Taabtenga (virtually devoid of infrastructural development) is on the periphery of the city and has rural characteristics. Each district has socio-cultural differences and differing health care facilities.

### Study design

The design adopted was pre- and post-intervention (conducted between 2006 and 2009) and were carried out in three phases. Phase 1 involved the baseline behavioural survey and community sensitization and mobilization, the selection of CMDs, and the determination of key baseline indicators. Information, education and communication (IEC) materials were developed and staff recruited and trained by staff of MoH and the research teams. Phase 2 was the intervention phase which involved the training of the CMDs and the distribution of ACTs according to the criteria for diagnosis and treatment, i.e. CMDs examining the children with fever (selection and training of CMDs are given in Table 2) and providing the caregivers with ACT and advice on how to administer it. Phase 3 was the evaluation phase, which was conducted a year after the commencement of Phase 2 by Health Centre staff and the various research teams, and lasted from one to two months

**Table 2:** Characteristics of the CMDs

Kumasi	Bolgatanga	Jimma	Lilongwe	Ouagadougou
35: 18 males and 17 females	28**: **18 females and 10 males	92: 60 females and 32 males	8: 2 males and 6 females	36: 30 females and 6 males
**Selection criteria: **ability to read and write; keep simple records; live in the community; trusted and willingness to volunteer	**Selection criteria:** Be stable; previous volunteer experience; considered responsible and reliable; have **b**asic education	**Selection criteria:** Member of Idir at least for last one year; willing to work voluntarily; available for at least 2 years; available most of the time; acceptable by the community	**Selection criteria: **Employees of the Ministry of Health. CMDs have secondary level education. Not resident in the community	**Selection criteria:** Resident; availability; ability to read and write French; acceptable to local people; accept to work for the community; and general interest of the community

**Main occupation:** Unemployed: 25% Petty trader: 25% Teacher/Social worker: 12.5% Artisan: 15% Local leaders: 10%	**Main occupation:** Drug shop owners: 35.7% Mason: 7.14% Petty traders 35.7% Farmers: 17.85% Retired nurse: 3.57%	**Main occupation:** Unemployed: 70% (housewife) Students: 12% Petty traders: 11% Daily labourers: 5% Retired: 2%	**Main occupation:** Full time employment as Ministry of Health CMDs	**Main occupation:** Unemployed: 54% (housewife) Petty traders: 11% Artisan: 17% worker: 12% Students: 6%

**Training:** Initial 5-day non-residential training. Monthly updates held in the first three months of the intervention and two-monthly updates	**Training:** 2 weeks training, conducted by the Regional Health Training Unit incl. two days at the Regional Health Laboratory	**Training:** Three day training. Training conducted in Amharic	**Training:** 6 weeks by MOH. Trained for two weeks on administering pre-packs: Week 1 theory and Week 2 practical	**Training:** Two day training conducted in each zone of the site.

**Supervision:** Fortnightly supervision by 2 members of the research team	**Supervision:** Weekly supervision, by 2 members of the research team, when data was collected from CMDs, plus monthly meeting of CMDs	**Supervision:** Monthly monitoring and supervision meetings were held. 10 supervisors (from Jimma Health Admin. Bureau, Jimma Zone Health Bureau and Jimma University Specialized Hospital) supervised the CMDs.	**Supervision:** Regular supervision (3 times weekly) by District Malaria Coordinator, plus monthly joint meetings held with community members, District Malaria Coordinator and research team	**Supervision:** Regular supervision of the each CMD by the Chief Nursing Officer of the health facility to which the CMD was attached

### Community medicine distributors (CMDs)

Although providing similar services in all sites, each of the five study sites described their CMDs differently: Bolgatanga had Community-based Drug distributers (CDDs); Kumasi had Community-based Agents or Community Medicine Distributors; Lilongwe had Health Surveillance Assistants (HSAs); Jimma had Community Health Volunteers (CHVs) and Ouagadougou had Agents de santé communautaire (ASCs). For the purpose of this paper all these are referred to as Community Medicine Distributors (CMDs).

A summary of the characteristics of the CMDs, including the selection criteria, is provided in Table 2. They were selected by their communities on the basis of them being members of the community, and acceptable to the members. In general they were volunteers who were trusted, could read and write, and were accessible to mothers and caregivers. Only in Malawi were CMDs full-time government employees who did not always originate from the communities where they worked. Here they are actually community health workers recruited by the Ministry of Health and paid by the Government of Malawi. CMDs in Lilongwe operated from a clinic-like setting during official working hours with patients and caregivers alike queuing for their services. Their normal role is to provide vaccinations and growth monitoring for < 5 year-olds; supervision of sanitation; water source protection and treatment; nutrition advice; disease surveillance, and provision of family planning services. In addition to these services, they were recruited into this study to presumptively diagnose and treat malaria cases in < 5 year-olds.

CMDs operating in the other sites were comparable as they were selected by the communities and had similar characteristics: they lived in the study communities; had at least a basic level of education; were volunteers; the majority were employed and did their CMD job as a secondary occupation; a few were unemployed, and they had varying occupational backgrounds (see Table 2).

### Sample size

In order to have an acceptable number of < 5 year-olds for the study, a sampling frame that encompassed a large enough urban area to provide a statistically significant number of householders for interviews and an acceptable number of malaria cases was used. In all study sites, the sampling frame comprised households (caregivers or mothers) with children aged 6-59 months in the study communities. As the main outcome measure for each study was the proportion of fever episodes treated adequately within 24 hours, and as this parameter was not known, a prevalence figure of 50% with a power of 90% at the 5% significance level and a design effect of 2 was used to calculate the sample size, with the exceptions of Bolgatanga, which used 10% (obtained from the literature as there were no recent surveys) and Ouagadougou which used 56% (derived from a previous study of caregivers). For interview purposes, a caregiver was selected by simple random sampling from each household. Thus each household was represented by a caregiver or mother with a child aged 6 months to five years of age.

The minimum number of households interviewed ranged from 794 to 956 per site.

### Data collection methods

A household survey was conducted in all study areas at the beginning of the investigation, to gather baseline data on the perception and practice of caregivers on their knowledge of signs and symptoms of malaria, and on their treatment seeking behaviour. This was repeated in all areas at the end of the evaluation phase. During this latter survey only caregivers whose children were febrile within the preceding two weeks were interviewed. The decision to admit children who had been febrile within the preceding two weeks was to assess those who required the CMD services within a reasonable recall period. In addition, at the end of the evaluation phase, a CMD register survey was undertaken to assess the CMDs performances in delivering ACT.

### Qualitative methods

Focus Group Discussions (FGDs) (253) and In-depth Interviews (IDIs) (217) were used to assess the acceptability of the CMDs providing ACTs. The discussions involved policy makers, implementers and community members, including caregivers. These tools were also used to assess the progress and opinions on the interventions, challenges and contextual issues. Discussions and interviews were held with men and women's groups, and community health personnel. Interviews were held with private and public health personnel, drug sellers, traditional healers, chemists/pharmacists, and community leaders.

### Medicines used in the studies

The national drug policies of the participating countries were adhered to. Thus, in Ghana artesunate and amodiaquine (AS/AQ) were prescribed, in combination, at the following dosage regimes: 25 mg/75 mg once daily for 3 days in children 6-11 months, and 50 mg/150 mg once daily for 3 days in children 12-59 months. In 2005, Burkina Faso adopted two ACTs (AL and AS/AQ) when the national policy for uncomplicated malaria treatment was changed. Both were considered "on an equal footing" and the policy decision to have two ACTs was made to prevent stock-outs should they have had only one ACT. Subsequently, the National Malaria Control Programme (NMCP) provided health facilities with AS/AQ more often than with AL. In the other countries artemether-lumefantrine (Coartem^®^) (AL) was prescribed at the following regimes: 20 mg/120 mg twice daily for 3 days in children 6-35 months, and 40 mg/240 mg twice daily for 3 days in children 36-59 months.

The AS/AQ used in Ghana and Burkina Faso was delivered as co-blistered packs for those children aged 12 months and older. However, the research team re-packaged and labelled the anti-malarials locally for those children aged 6-11 months after having cut the tablets in half because, at the time of the study, there were no commercially pre-packed anti-malarials available for that age group of the appropriate strength. The AL used in the other countries was provided as co-formulated medicine pre-packed by the manufacturer.

In accordance with national policies, anti-malarial drugs were provided for free in Malawi and Ethiopia and against payment of a small amount of money in Burkina Faso and Ghana. In Burkina Faso, the amount charged to patients was the same at health centre and community level; CMDs, however, were allowed to keep 10% of the sales revenues as an incentive for their work when they were replenishing their stock at the health centre.

Details of the supply of medicines for each study group (including costs) are given in Table 3.

**Table 3:** Supply of Medicines

Kumasi	Bolgatanga	Jimma	Lilongwe	Ouagadougou
ACTs (AS/AQ) procured by the MOH	ACTs (AS/AQ) supplied by UNICEF	Jimma town health bureau procured ACTs (AL) from Oromiya Regional Health Bureau	ACTs (AL) supplied by MOH	ACTs supplied by WHO to MEG. 4334 units of ACTs (AL) then made available for sale and return to 21 public/private drug seller and CMDs

All CMDs were given two week supplies	Weekly supervision for each CMD to supply and collect cases seen weekly	Stocks kept at Jimma Health Centre	The study team was responsible for maintaining stocks at the community at regular supervision meetings	Stocks were kept at CNRFP and CMDs were given supplies when needed

ACTs sold at GH¢0.50 (US$0.455)* for preschool children and GH¢0.20 (US$0.182)* for infants	ACTS sold at GH¢0.15 (US$0.137)*	Treatment was free	Treatment was free	ACTs sold at 100 F CFA (US$0.23)** for children: 6 months-3 years and 150 F CFA (US$0.34)** for children: 3-5 years

Medicines not used after two weeks were withdrawn as a quality control measure	Stock-out only for paracetamol	ACTs after expiry date not used, and withdrawn as quality control measure	Drug stocks were replenished on a monthly basis	Drug stocks were replenished on a monthly basis

*(GH¢1 = USD0.91 (2007)) ** (FCFA 1 = USD0.0023)

### Supervision

CMDs were regularly supervised in all countries according to national policies. In Kumasi, the CMDs were supervised on a fortnightly basis by the research team members, whilst in Bolgatanga there was weekly supervision by members of the research team. During each visit the research sought to know from each CMD, when supplies of ACT were distributed and the numbers of malaria cases recorded. In addition, monthly CMD meetings were held. In Jimma there were monthly monitoring and supervisory meetings conducted by the research team. The supervisors were drawn from the Jimma Health Administration Bureau, Jimma Zone Health Bureau and the Jimma University Specialized Hospital. In Lilongwe there was regular supervision (three times a week) by the District Malaria Coordinator, plus monthly joint meetings with community members, the District Malaria Coordinator and the research team. In Ouagadougou supervision was carried out weekly by the chief nursing officer of the health facility to which the CMD was attached and monthly by the research team.

### Data analysis

Epi Info version 6.02, SPSS version 11.0 and STATA version 9.2 were used by the study groups for entering and analysing the data. A set of indicators (apply to corresponding author for a copy) and quantitative analyses to be performed were agreed in discussion between the study teams and the TDR facilitators in a meeting held in Nairobi in 2005. Each research team then undertook the analyses of their own data.

The FGDs and the IDI information was tape-recorded, transcribed, categorized and analysed according to study themes at each country level by the research teams. The analyses were conducted manually.

### Ethical approval

Ethical approval for all these studies was granted by the WHO Ethics Review Committee and by the appropriate national and Institutional Ethical Review Boards of each participating country. Informed consent was obtained from community leaders, household heads and the caregivers who participated in the studies.

## Results

### Community medicine distributors in urban CCMm

The ratio of female to male CMDs varied from 1:1 (Kumasi) to 5:1 (Ouagadougou). The average number of children that each CMD had under his/her care varied from 55 in Ouagadougou to 570 in Lilongwe. The differences are mainly due to different population densities and how scattered the settlements were in the various areas. In several cases, CMDs had commercial outlets not related to health (e.g. barber or video shops) or were teachers.

Housewives/unemployed were the most common CMDs followed by traders. Interestingly, in Bolgatanga drug shop owners were the most common CMDs along with traders. Artisans, labourers, farmers and students made up the bulk of the rest of CMD occupations in each of the study areas. Overall, it is interesting to note the much greater variety of cadres on which urban CCMm programmes can draw compared to rural CCMm ones. They were available to serve as CMDs because this did not interfere with their main job, and sometimes it would even bring them a commercial benefit. In rural CCMm programmes, on the contrary, where the majority of CMDs are subsistence farmers, serving as a CMD at times interferes heavily with their personal commitments.

Overall, the use of services by CMDs was high. A total of 9001 children with an episode of fever were treated by 199 CMDs in the five study sites during the study period, with the average number of children seen by each CMD varying between 27 and 266 (average 45) (Table 4). Demand for the services was positively related to having a primary education level and the source of money for health care.

**Table 4:** Measures of CMD utilization and performance in delivering ACTs (source: CMD registers)

	Kumasi	Bolgatanga	Jimma	Lilongwe	Ouagadougou	Total
Number of CMDs	35	28	92	8	36	199
Total number of febrile children seen by CMDs (average per CMD)	955 (27)	1,363 (49)	2,634 (29)	2,131(266)	1,918 (53)	9,001 (45)

Duration of Intervention Phase Ave. no. febrile children seen per month Ave. no. febrile children seen per CMD per month	12 months 80 2.3	12 months 114 4	24 months 110 1.2		15 months 128 3.5	

Number of children treated with ACTs - N (%) within 24 hrs - N (%) treated with the correct dose	905 (94.8)** 577 (63.76) 890 (98.3)	909 (66.7)* 278 (31%) 888 (97.7)	1,211 (46)* 784 (64.6%) 1,206 (99.6)	2,131 (100)** Not available 2,131 (100)	1,818 (95%)** Not available 1,818 (100)	6,974 1639 (54.2) 6933 (99.4)

* Treatment based on RDT result

** Treatment based on presumptive diagnosis.

### Perceptions and practice

Two household surveys were held to measure the caregivers' knowledge of signs and symptoms of malaria and also on their treatment seeking behaviour. The first survey was implemented at the beginning of the investigation and the second at the end of the evaluation phase. The results are shown in Table 5.

**Table 5:** Perception and practice (source HH survey)

Indicators	Kumasi		Bolgatanga		Jimma		Lilongwe		Ouagadougou	
	
	Base (N = 1054)	End (N = 1000)	Base (N = 530)	End (N = 956)	Base (N = 823)	End (N = 2,307)	Base (N = 823)	End (N = 839)	Base (N = 625)	End (N = 798)
**1. % knowledge of signs and symptoms of severe malaria**										
■ Vomiting	3.8	42.7	9.9	3.9	59.4	24.9	12.8	61.5	36.8	46
■ Diarrhoea	5.7	59.8	14.5	41.6	6.6	1.6	9	36.1	12.3	32
■ Loss of consciousness	3.2	0.7	2.8	1.2	-	-	44.1	29.0	18.3	14
■ Convulsions	8.0	4.3	-	-	65.6	34.8	43.3	59.2	22.0	31
■ Yellowness	8.0	42.7	-	-	-	-	5.2	20.4	26.0	21
■ High body temperature	50.7	71.7	35.5	14.9	96.2	85.5	0.7	2.6	70.2	95
■ Inability to feed/suckle	11.6	22.2	4.5	4.8	44.0	33.2	27.5	69.7	25.0	55
■ Weakness	40.0	0	43.8	32.1	5.6	6.1	10.5	18.6	26.0	27
■ Not passing urine	1.1	34.2	-	-	0	-	22.0	53.4	-	-
■ Fast breathing	16.8	23.7	-	-	-	-	17.4	20.7	-	-
■ Drowsy	3.7	61.7	-	-	-	-	3.4	35.6	7.1	-
■ Don't know	7.1		-	-	-	-	26.0	7.2	-	-
■ Other	14.0		0.9	1.5	-	-	0	3.2	-	-

**2. Preferred provider for childhood malaria treatment**	**(N =** **1054)**	**(N =** **1000)**	**(N =** **930)**	**(N =** **956)**	**(N = 823)**	**(N = 2305)**	**(N =** **671)**	**(N =** **839)**	**(N =** **625)**	**(N =** **798)**
■ Self-treatment (herbs)	0.0	0.0	30	0.6	0	0	6.6	3.0	29	13
■ Self Treatment (mod. med.)	0.0	53.2	-	5.7	0.0	5.6	68.1	26.6	-	3.4
■ CMDs	6.8	31.1	-	61.2	74.7	82.7	48.4	64.2	54	57
■ Public health facility	9.2	0.0	47.18	28.2	24.2	10.8	21.6	93.9	--	--
■ Private clinics	0.0	0.9	7.97	0.4	0.1	0.3	1.9	6.4	4	2
■ Traditional healers	2.5	0.0	0.44	0.4	0.8	0.3	1.0	0.5	5	4
■ Informal medicine vendors	3.1	24.3	10.52	0.8	0	0	47.5	5.1	23	29
■ Chemists/pharmacists	4.8	2.0	3.65	2.7	-	-	0	1.5	**-**	**-**

In both Bolgatanga and Jimma fewer caregivers reported gaining no knowledge over the period of assessment (Table 5) as opposed to the other three sites where there were overall improvements in their knowledge of signs and symptoms during the same period.

Regarding the preferred providers of malaria treatment, all sites reported an improvement in preference for CMDs. The highest percentage change was seen in Bolgatanga (61.2%), followed by Kumasi (24.3% change), Lilongwe (15.8% change) and then Jimma (8%) and Ouagadougou (3%). The use of modern medicines for self-treatment increased in four of five sites, while the use of public health facilities decreased, and the use of traditional healers either declined or remained static.

No use of informal medicine vendors was made in Jimma. The preferred use of chemists/pharmacists declined in Bolgatanga and Kumasi, and increased in Lilongwe. Their use was not recorded in Jimma or Ouagadougou.

### Measures of performance

The performance of CMDs was evaluated on the quality of their delivery of ACTs (Table 5). In all sites a total of 9,001 febrile children were seen by CMDs. The number of febrile children seen, on average, by each CMD per month during the evaluation phase varied from 1.2 (Jimma) to 4 (Bolgatanga); excluding Lilongwe where the operational conditions and work practices were different. Of these 9,001 febrile children, 6,974 (77.5%) were diagnosed as having malaria, and were treated with ACT; and 6,933 children (99.4%) were treated with the correct dose. Information about the promptness of treatment was available for 3,025 children; 54.2% of them were recorded as having had their treatment initiated within 24 hours of onset of symptoms.

### Indicators of treatment coverage

As an indicator of treatment coverage by the CMDs a household survey was conducted. The recall period for this survey was two weeks (Table 6). The total number of children with a fever episode within the two-week period of the survey was 3,700 but only 1,480 (40%) of caregivers consulted a CMD as the first point of call. Of these 1,480 children, 1,213 (82%) were treated with an ACT, 1,123 (92.6%) of them with the correct number of doses and for the correct number of days, and 828 (68.3%) were reported to have been treated within 24 hours of the onset of symptoms. Overall, 820 children (67.6%) were treated correctly and promptly.

**Table 6:** Indicators of treatment coverage with ACTs obtained from CMDs and of adherence of caregivers to treatment schedule (source: household survey)

	Kumasi	Bolgatanga	Jimma	Lilongwe	Ouagadougou	Total
Total number of children with a fever episode in the previous 2 weeks identified at survey	433	542	689	1238	798	3700

• Number (%) of caregivers who consulted the CMD	179 (41.1)	168 (31)	50 (7.3)	801 (64.7)	282 (35.3)	1480 (40)

• Number (%) of children treated with ACTs from a CMD	179 (100)**	106 (63.) *	32 (64)*	801 (100)**	95 (34) **	1213 (82)

• Number (%) of children correctly treated (dose and duration)	167 (93.1)	53 (50)	32 (100)	798 (99, 6)	73 (76)	1123 (92.6)

• Number (%) of children treated promptly (receiving first dose within24 hrs)	123 (68, 7)	37 (34.9)	23 (71.9)	590 (73, 7)	55 (57.9)	828 (68.3)

• Number (%) of children treated correctly AND promptly	123 (68, 7)	37 (34.9)	18 (56.3)/32	590 (73, 7)	52 (54.7)	820 (67.6)

• Number (%) of mothers who did not find the CMD at the first attempt	4 (2.2)	Not available	7 (14)	Not available	36 (12.7)	

* Treatment based on RDT result

** Treatment based on presumptive diagnosis.

### Qualitative findings

A total of 253 FGDs and 217 IDIs were carried out during the baseline and evaluation phases. The majority of FGDs (187) were conducted with mothers and 79 IDI were conducted with community leaders. Overall, all sectors of the communities were addressed: health workers, public health service providers, health managers, pharmacists, NGOs, community health workers and practitioners. Most of the FGDs were conducted in Ouagadougou and the majority of IDIs were conducted in Jimma. There was considerable variation between the five study sites in the number of qualitative investigations carried out, and there was no consistency between the groups.

### People's perceptions as captured through the qualitative study tools

The findings were discussed during the report production workshop, and were interpreted and presented in the form of narratives comparing the results across the sites. The thematic narrative findings of the FGDs and the IDIs are presented below.

#### Perceptions on malaria

The qualitative data indicated that many respondents knew about the signs and symptoms of severe malaria. The most commonly cited symptoms were vomiting, high body temperature and inability to feed.

A participant in Kumasi noted that:

*Affected children may be convulsive, have high body temperature, refuse to accept and eat any food, sometimes vomit virtually all food and water out, vomit is sometimes yellowish (Kumasi, Ghana)*.

Most participants, through the FGDs, mentioned that the major sources of information were the CMDs, radios, local community health providers, hospitals and friends.

#### Health-seeking behaviour

In Malawi, most participants indicated that they used the HSA centres. This was mainly due to the fact that there were no other formal health providers in Kauma before the introduction of the Centres. In Bolgatanga, a participant in a female FGD stated that:

*There are some women in our community who have been chosen and trained. If you are near one of them you can send your child there. They have something with which they test the children before they give them the medicine. If the child has malaria they will give the medicine *(Bolgatanga, Ghana).

The following sequence in health seeking was reported more generally, with some minor variations in the five sites:

(i) caregivers seek the opinion of spouses, in-laws and/or elderly relatives;

(ii) children are sponged and/or given available medication (including orthodox and herbal preparations);

(iii) medicines are bought from chemists/shops;

and (iv) that care is sought subject to affordability, recommendations of friends or relatives and prior experience of the caregiver.

#### What determines choice of provider?

The majority of the participants, in both the FGDs and IDIs, indicated that the severity of the illness determined the choice of the provider.

A caregiver in Lilongwe observed that:

*If the child is serious we do not wait for anything, we just take the child to the health centre, regardless of time *(Lilongwe, Malawi).

#### Views towards pre-packs

The pre-packs were generally accepted and described as very effective for treating fever, available all the time, cheaper and affordable. In addition they considered the pre-packs a helpful strategy. In Ghana, it was notable that most participants were happy with the intervention and they wished it could cover everyone in the community not just children. A participant in Bolgatanga stated that

*It is a malaria drug, if you are going to give this drug to a child like this, you will break the yellow tablet into two and then break it into two again. You do the same thing to the white one. Then you dissolve the drugs on a spoon with water and give to your child to drink *(Bolgatanga, Ghana).

There was an indication that the caregivers had acquired adequate knowledge on the drug regimen. Some remembered the colours of the drugs but were not certain about the dosage and the number of days for drug administration. Although the mothers were expected to return to the CMDs, it was noted that this was difficult due to their engagement in income generating activities. Some of those who failed to comply stated that it was due to the immediate recovery of the child after taking the first dose.

The positive view across the countries on CMD as a channel of distribution was mainly due to proximity. The terms used to describe CMDs included: appropriate, comparatively stress-free, convenient and preferable source. The participants in Ghana expressed the view that complaints and the need for further explanation were addressed promptly by CMDs, and CMDs occasionally visited the mothers in their homes.

There were, however, some community members who did not appreciate the work of the CMDs. This was mainly related to perceptions towards their low skill levels. A CMD in Bolgatanga reported the following:

*They say they don't know where we have gone for training that has given us the knowledge to treat children. That person told me he does not like our medicine. He said he would rather take his child to hospital. He does not know the school I attended and the training to enable me sell medicines in the community *(Bolgatanga, Ghana).

In Ethiopia, although the CMDs were known to mothers in Hirmata Markato, their services did not attract the attention of most households. Thus, during malaria illness, mothers often took their children to the health centre than seek the help of CMDs. The reasons include the fact that mothers were already conditioned to the health centre or they lacked confidence with the CMDs. Reasons for preference for private facilities included: to get prompt diagnosis and hence timely treatment; private clinics were nearby in the community; and private clinics felt more responsibile and were more accountable than public health care settings.

#### Barriers and challenges

The study highlighted some challenges experienced in the delivery of treatment to children. In Malawi, the main issue was the availability of the CMDs at all times. This was mainly due to the fact that most of them were not resident in the area and, therefore, their hours of operation were limited from 10.00 to 16.00 hours.

A major problem in the study sites was poverty given that the selected communities were in urban poor areas. In Ethiopia, a participant noted:

*We are living in abject poverty. Thus, financial insecurity has curtailed our ability to buy drugs, food items and meet other extra costs when our children are ill *(Jimma, Ethiopia).

In Ouagadougou, staff at health facilities thought stocks of Coartem were exhausted very quickly because of competition between CMDs and health centres:

*There was no regulation which required us to keep some stocks especially for CMDs. Now, people come to the HC from everywhere and not just Taabtenga and Wemtenga. When we diagnosed malaria we prescribed Coartem, which they could find cheaper here *(a nurse from Wemtenga health facility). If there was a stock-out of ACs in the pharmacies, then the public attended the HCs where they were provided with anti-malarials.

## Discussion

Until relatively recently malaria has always been considered a rural disease in Africa mainly because of the perception that suitable malaria vector breeding sites in towns and cities were rare. This may explain why the transmission of malaria is generally lower in the urban environments, when compared to that of rural areas; and together with a greater availability of health care providers and a generally higher socio-economic status in the urban situation [18] results in a relatively lower malaria morbidity and mortality [19]. However, data from Accra and Kumasi, Ghana demonstrated that malaria can be a major public health problem in the urban setting and that prevalence can vary markedly between communities and between cities [20].

The studies described in this paper are implementation research studies, which (by definition) have to follow national policies and be embedded in 'real life' local ways and cultures. This accounts for the diverse type of CMDs operating in different countries ('traditional CHWs in some countries vs. HSA on the government payroll in Malawi) and may explain either the diversity of the effects observed on health seeking behaviour and some of the differences in performance or impact between studies.

### Community medicine distributers in urban CCMm

Notwithstanding differences between sites, the results show that CCMm in African urban environments is a feasible option, which is well accepted by the populations. In line with findings in rural areas, the overall quality of care provided by CMDs is quite satisfactory (99.2% of correct prescriptions), as well as adherence by caregivers to the correct treatment schedule (92.6%) and the promptness of administering the first dose (68.3%). However, the coverage obtained by urban CCMm is generally more modest than in rural environments, with "only" 40% of caregivers consulting CMDs for the treatment of their febrile children compared to the overall figure of 59% noted by Ajayi [16].

In Jimma, about 80% of caregivers reported that their preferred provider for childhood malaria treatment was the CMDs because services were available in the neighbourhood and were quickly performed, however only 7.3% of caregivers used the services as they preferred to utilize the 'established' health services. In fact, this apparent disconnect between perceptions and practice was also evident in Bolgatanga and Ouagadougou, although the differences were not as great as in Jimma. In Kumasi, only 31% said that they would prefer to use the services of the CMDs, but actually 41% used them. These differences between perceptions and practice can generally be accounted for by caregivers' preference for the existing systems (which they know); the proximity of established pharmacies, clinics, etc.; lacking confidence in their CMD; not finding the CMD at the first attempt; professing to be unaware of the quality of their training; stock-outs, and inability to pay. Of interest is the situation in Lilongwe, where 64% of respondents said they would prefer the CMDs, and the same proportion actually used them. The point about the study area in Lilongwe (Kauma) is that the CMDs were the only health service providers, and as has been stated previously, the eight CMDs had a much wider role than those in the other sites under investigation. The fact that the Lilongwe CMDs had, in addition to their normal duties, malaria diagnosis and treatment, meant that they were filling a void in the area. Thus it is not surprising that more people sought their services than going elsewhere.

The main reason that CMDs were used was because of proximity; they were trained, and they provided quick treatment.

It would appear that the effectiveness of the CCMm is directly linked to the efforts of the CMDs to become familiar with the caregivers in their areas, and to engender trust in their ability to diagnose and treat childhood malaria quickly, safely and effectively.

### Perceptions and practice; measures of performance and indicators of treatment coverage

A comparison of those parameters that were measured by both the household survey and the CMDs registers shows a close agreement between what the CMDs recorded and that which the caregivers recalled in the last two weeks of the implementation phase in terms of quality of care and utilization of services.

The CMD registers showed that 99.4% of the children were treated with the correct dose for their age, and the household survey showed that 92.6% were treated with the correct dose and for the correct number of days.

The CMDs recorded that 54.2% of the children were treated within 24 hours of being ill, whereas the household survey recorded 67.6%. We acknowledge that the data are not strictly comparable, as CMD registers were maintained over a period of one year (the intervention phase) and the household survey was conducted at the end of the intervention phase with a recall period of two weeks. In a similar study carried out in rural areas of sub-Saharan Africa [16] adherence was measured in terms of caregivers' report of the number of doses administered to the child, the number of days over which treatment was given and the promptness of treatment after the onset of symptoms. Overall 85% of children were treated correctly in terms of drug dose and duration of administration. This figure is in line with those of the present study in urban communities, where the overall adherence was 92.6% and greater than 90% in three of the five sites.

The unit dosed pre-packed anti-malarial medication for the CCMm is widely accepted amongst mothers [21] and this study found over 90% and 84% acceptable levels for the use of pre-packs at the baseline and after the intervention. Willingness to pay for pre-packs amongst caregivers was slightly over 50%. In Bolgatanga, a moderate price (US$0.137) was affordable to most caregivers and as it was much lower than the prevailing price at the municipal health facility it was highly acceptable. This was cheaper than the prices charged for ACT in Kumasi (US$0.182 and US$0.455) or in Ouagadougou (US$0.23 and US$0.34), where the costs were acceptable. A future debate may therefore revolve around the affordable charges that programmes could make for the confirmatory diagnosis and treatment of malaria. Given the figures presented here, it is possible to conjecture that diagnosis may cost US$1.00 [22] and ACT up to US$0.50, making a charge of US$1.50 per malaria confirmed under-five year old child. The question then becomes "*Is CCMm affordable in the urban setting, or would caregivers find it cheaper to seek care in the private sector?" *Self-treatment continues to be common, and in urban areas it is commonly due to the large numbers of medicine vendors (formal and informal) that exist. Various reasons why people patronize drug shops, pharmacies, and even illegal drug sellers instead of health centres and hospitals, indicate that it is the ease of buying and obtaining immediate treatment that encourages them. In a study in Maiduguri in Nigeria, it was reported that immediate attention for both consultations and treatment was the most important reason why pharmacies and drug shops were frequently patronized, rather than the health centres [23]. Thus, it would appear that self-treatment is a common practice in malaria endemic regions of Africa, and this may be even more prevalent in urban areas. A recent study in urban Kampala in which anti-malarial drugs were stored by mothers at home and given to children if they developed fever, showed that there was substantial over-treatment and little effect on clinical outcome [24]. While we agree with the authors that ACT provided *in the home *might not be appropriate for large urban areas or settings with fairly low malaria transmission, the findings of our study show that when drugs are distributed by appropriately trained CMDs, the quality of prescription and adherence to treatment schedules by caregivers are as high as in rural areas with high malaria transmission.

### Qualitative findings

Qualitative research findings of the study indicate that the acceptability of pre-packed ACTs for treating malaria in children under 5 was very high. Compliance with the recommended dosage, as well as treating children with malaria or fever within 24 hours by caregivers was said to have improved significantly across sites. These data indicate that the use of the CCMm in urban settings is feasible and acceptable to caregivers, and contributes to an improvement of malaria case management.

It is vital for the success of CCMm in urban situations to implement an effective and sustained health educational programme aimed at caregivers, coupled with an equally important educational and training programme specifically for health personnel. It was extremely difficult to mobilize urban communities for health education programmes. In Kumasi, only about 25% of caregivers sought care for their children either from clinics or hospitals depending on distance and recommendations from relatives and friends (Kumasi interviewed respondents).

The main challenge to urban CCMm is the motivation of CMDs in order to sustain the pre-packed ACTs [25]. At this stage it is not possible to state whether it was a failing of the IEC programmes, socio-cultural barriers or that it was the 'distraction' of competing health care providers that seemingly prevented a higher utilisation rate of CMDs. IEC strategies that are effective in rural settings may not be appropriate in urban environments, and sensitization programmes should be tailored to the urban setting. The experience gathered in these studies shows, for instance, that most IEC messages conveyed through local radio and television are more effective than through the traditional use of religious meetings, which has the highest impact in rural communities.

Considering that the IEC campaigns were not as successful as anticipated, did the caregivers gain any knowledge about malaria signs and symptoms during the period of the intervention? In Kumasi, Lilongwe and Ouagadougou there were marked improvements (Table 4). In Lilongwe there was an improvement of knowledge of nearly all the signs and symptoms listed; in Kumasi fewer signs and symptoms were listed as showing some improvement and in Ouagadougou there were less still. In Lilongwe and Ouagadougou the important signs: vomiting, convulsions, inability to feed/suckle and high temperature showed marked improvement, whilst in Kumasi knowledge of vomiting, high temperature and inability to feed/suckle showed marked improvement. In both Bolgatanga and Jimma, the caregivers tended to lose knowledge, with only one or two signs and symptoms showing any improvement.

CMDs will not always work as volunteers. They need remuneration or incentives in order for them to make an intervention sustainable and effective, e.g. in the Sudan the volunteer Malaria Control Assistants worked on a consultation fee of US$0.50 [26] and in the Ouagadougou study reported here, the vendors of ACT were allowed to keep 10% of the sales as a personal incentive. In order for the CCMm approach to be valued in urban situations, the concept should be supported by the local health personnel, who would then see the CMDs as official health workers who spearhead an outreach service.

By using collaborative approaches that include the community, the private sector and existing health, urban planning, agricultural and governance structures, urban malaria is uniquely amenable to prevention and control [27].

## Conclusions

Most of the FGD participants appreciated the project and acknowledged the approach adopted. They observed that being served or getting malaria treatment closer to their homes was highly valued and something that everyone required to effectively manage childhood malaria.

The concept of CCMm in the urban environment was positive, and those caregivers who used the system were generally satisfied. Overall, 40% of caregivers opted for their children with fever to be treated by CMDs, and the vast majority of them were treated promptly and correctly. This shows that CCMm has an important role to play as a public health strategy for malaria case management in urban as well as in rural settings. However, it is obvious that as a new system, it was not universally utilized, and there are several reasons for this. For example, it was generally considered that the IEC system was not nearly good enough, and the systems used in rural areas did not work in the cities. It also seems probable that some of the CMDs did not integrate well with the communities they served, thus jeopardizing the overall uptake of the system. In part, this may have been due to the fact that some form of incentive was a necessity to maintain the activities of the CMDs. The main reason for not using the system more was because there were traditional conflicting sources of health care, and the introduction of a novel system requires considerable input from the initiators as well as the community incumbents. It is probable that the need for incentives is higher in urban areas given that access to food and all services is cash based.

Incentives for CMDs are an important aspect of the development of this system of delivering malaria diagnosis and treatment. It is recommended that RDTs be provided to CMDs for improved diagnosis of malaria, in keeping with WHOs latest edict [28].

Urban CCMm is feasible, but faces an uphill battle compared to its rural counterpart. Within the urban context there are many competing health outlets, all of which have been serving the communities for many years. The introduction of a new system requires time and patience for the community to evaluate it, and to accept it as an improved system to that which currently supplies their needs. Since there are many conflicting sources of medicines in urban centres it may also be possible to integrate CCMm with private health care providers, as long as the advantages of cost, good advice and CMDs being "friends of the households" are maintained. The programmes must, therefore, invest in community mobilization to market the intervention.

## Competing interests

The authors declare that they have no competing interests.

## Authors' contributions

All the authors except GB conceived the study; MS, ATK, PC, PA-B and PA were principal investigators for their respective country's study site and together with PA, AT, BS and ELNB participated in the research design and supervised data collection from the field. Apart from the PIs, Samuel Boateng (Ghana), AT (Jimma) and BS (Malawi) performed most of the quantitative data analysis and analysed the qualitative data. FP, the WHO/TDR CCMm research programme manager supervised all the field sites. FP, MA-N and GB facilitated the data analysis workshop. GB co-ordinated all the final reports and developed the final manuscript. All authors read and approved the final manuscript.
